# Collective violence and the health of the elderly: a cross-sectional analysis of a population-based national survey in Mexico

**DOI:** 10.26633/RPSP.2017.29

**Published:** 2017-03-22

**Authors:** Carmen García-Peña,, Natalia Sánchez-Garrido, Emma Grace Wynne-Bannister, Bernardo Moreno-Peniche, Mario Ulises Pérez-Zepeda

**Affiliations:** 1 Research Office National Institute of Geriatrics Mexico City Mexico Research Office, National Institute of Geriatrics, Mexico City, Mexico; 2 Department of Geriatric and Epidemiologic Research National Institute of Geriatrics Mexico City Mexico Department of Geriatric and Epidemiologic Research, National Institute of Geriatrics, Mexico City, Mexico.; 3 School of Medicine National Autonomous University of Mexico Mexico City Mexico Department of Geriatric and Epidemiologic Research, National Institute of Geriatrics, Mexico City, Mexico.

**Keywords:** Aging, violence, exposure to violence, health of the elderly, Mexico, Envejecimiento, violencia, exposición a la violencia, salud del anciano, México, Envelhecimento, violência, exposição à violência, saúde do idoso, México

## Abstract

**Objective.:**

To describe the association between collective violence and the health of older adults in Mexico.

**Methods.:**

The data analyzed were taken from a Mexican population-based national survey of health and nutrition that included a representative sample of adults over 60 years of age and from an index of violence for each of the states of Mexico that was compiled by a major research center. Five of the most common geriatric ailments (weight loss, depressive symptoms, falls, positive affectivity, and disability) were crossed with the violence index score assigned to each state.

**Results.:**

A total of 7 108 older adults were included in the analysis. Among the five geriatric health problems, weight loss had the strongest association with violence, even when an adjusted model was used. For weight loss, that association increased as the level of collective violence rose. With the adjusted model, there was also an association of severe collective violence with disability and with low positive affectivity.

**Conclusions.:**

Our results show that there is an association of collective violence with weight loss and other geriatric problems. Collective violence could indirectly affect individuals’ health, especially older persons and other vulnerable groups.

## RESUMEN

***Objetivo*.:**

Describir la asociación entre la violencia colectiva y la salud de los adultos mayores en México.

***Métodos*.:**

Los datos analizados se tomaron de una encuesta nacional de salud y nutrición de la población mexicana que incluía una muestra representativa de adultos mayores de 60 años y de un índice de violencia para cada uno de los estados de México, compilado por un importante centro de investigación. Se cruzaron cinco de las dolencias geriátricas más frecuentes (pérdida de peso, síntomas depresivos, caídas, afectividad positiva baja y discapacidad) con la puntuación del índice de violencia de cada estado.

En el análisis se incluyó un total de 7 108 adultos mayores. De los cinco problemas de salud geriátricos, la pérdida de peso reveló la asociación más fuerte con la violencia, incluso cuando se utilizó un modelo ajustado. Esa asociación aumentaba a medida que se elevaba el nivel de violencia colectiva. Con el modelo ajustado también se observó una asociación de la violencia colectiva grave con la discapacidad y con una afectividad positiva baja.

**Conclusiones.:**

Nuestros resultados indican que existe una asociación entre la violencia colectiva y la pérdida de peso y otros problemas geriátricos. La violencia colectiva podría afectar indirectamente la salud de las personas, especialmente los adultos mayores y las personas de otros grupos vulnerables.

## RESUMO

Descrever a associação entre a violência coletiva e a saúde do idoso no México.

Os dados analisados foram obtidos de um inquérito nacional de base populacional de saúde e nutrição conduzido no México com uma amostra representativa da população adulta acima de 60 anos de idade e com base em estimativas dos índices de violência dos estados mexicanos fornecidas por um grande centro de pesquisa. Cinco problemas geriátricos mais comuns (perda de peso, sintomas depressivos, quedas, afetividade positiva e incapacidade) foram examinados em relação ao índice de violência de cada estado.

A análise incluiu uma amostra de 7.108 idosos. Dentre os cinco problemas geriátricos, a perda de peso teve a associação mais expressiva com a violência, mesmo após o uso de um modelo ajustado. Esta associação aumentou com o nível de violência coletiva. No modelo ajustado, verificou-se também a associação de alto nível de violência coletiva com incapacidade e baixa afetividade positiva.

***Conclusões*.:**

Este estudo demonstra existir uma associação entre violência coletiva e perda de peso e outros problemas geriátricos. A violência coletiva possivelmente exerceria uma influência indireta na saúde da população, sobretudo de idosos e outros grupos vulneráveis.

Violence is defined by the World Health Organization (WHO) as “the intentional use of physical force or power, threatened or actual, against oneself, or against a group or community that either results in or has a high likelihood of resulting in injury, death, psychological harm, underdevelopment or deprivation” (1). A recent report on violence prevention highlights that there is impact on both psychological and physical health, and that more than 1.3 million people worldwide die each year as a result of violence (self-directed, interpersonal, and collective), accounting for 2.5% of global mortality (2). Although violence is considered a public health issue (3, 4), a recent work by Gamlin focusing on the current situation of Mexico and organized crime there urged the global health community to address the issue of violence, due to its magnitude and social implications (5).

The Government of Mexico declared a war on drugs in December 2006. Since then, thousands of people have died as a direct consequence of the measures that have been taken (6). As stated in a research study done by investigators at the University of Guadalajara (which is in Jalisco, a state with high rates of violence), violence is a social epidemic that is not only visible in death rates, but has also penetrated daily life and harmed the population's health and quality of life (7). Moreover, violence has continued growing under the current Government (which came into power in December, 2012), as shown in recent reports by nongovernmental organizations and as covered by the international press (8). This situation is what we call collective violence, and it is the type of violence that we researched for this study.

There is evidence that certain health issues worsen in violent environments, including domestic violence, as with abuse of older adults (9), or with collective violence, as with earlier menarche in Colombian girls exposed to violent environments (10). Collective violence has been thought to affect younger adults the most, but there is also a resulting overall disruption of social and family dynamics, which are impacted indirectly by crimes such as murder, kidnapping, and extortion (4, 11).

The fastest growing segment of the population in Mexico, as in other countries, is older adults (12), and interest is rising in how socioeconomic determinants can shape the health of this age group (13). The effect of collective violence on the health of the elderly has been poorly studied. It is highly likely that some geriatric ailments, such as disability, weight loss, falls, depressive symptoms, and low positive affectivity, could be present or worsen in a violent environment. A useful frame to approach this phenomenon is the so-called “explanatory context,” as Franco has defined it: a specific combination of cultural, economic, sociopolitical, and legal conditions that make a phenomenon possible and that provide for a description of the origin and explanation of a phenomenon (11). In this context, the complex health conditions of older adults that frequently produce a higher rate of health services utilization are, in fact, multicausal. Among the causes are the poor response of social and health systems and inequity in the distribution of resources (14, 15). For example, in Mexico the national budget for public health had an overall growth of 4.6% between 2003 and 2009, while the public security program’s budget increased 19.2% between 2001 and 2010 (16, 17).

There is a direct threat to life caused by collective violence. In addition, there are indirect consequences in terms of the mortality and morbidity coming from that violence, such as unstable health systems and deficient medical care (5, 11). For instance, in Mexico there has been a decrease in the number of primary care doctors in rural areas since the war on drugs started. Final-year medical students typically provide health care in rural areas during their compulsory year of social service. Due to violence, work conditions for these students have worsened, causing them to leave their posts or stay in urban areas to avoid violence (18, 19). This affects elders’ health by diminishing the already scarce health services in rural areas. Rural areas have a substantial portion of the older population: 26.22% of elders live in communities with fewer than 2 500 residents (20). In terms of health care services, these areas are also the ones with the lowest number of doctors per 1 000 inhabitants (21). These data add to the possible explanatory contexts that impact older adults’ health in such rarely-assessed areas as geriatric syndromes, cognitive decline, and frailty (22).

Therefore, it is possible to hypothesize that as part of the explanatory context, ongoing collective violence impacts the health of older adults. The aim of this work is to assess and describe the association between collective violence and the health of older adults in Mexico.

## MATERIALS AND METHODS

### Design and settings

This is a secondary cross-sectional analysis of Mexico’s ENSANUT 2012 (*Encuesta Nacional de Salud y Nutrici*ó*n* [National Survey on Health and Nutrition]), of which a detailed description is available elsewhere (23). In brief, this is the latest version of a nationwide survey that uses a household survey to gather data on health and health services utilization by children, adults, and older persons. The survey is repeated every six years. The data reported in this article are from the 2012 survey, which included 96 031 subjects from all over the country. A representative subsample answered specific questions on and underwent tests of particular interest for older adults’ health, including depressive symptoms, activities of daily living, cognition, falls, and physical performance (walking speed). With respect to the sample of older adults, after a probabilistic sampling by regions of the country, among a total of 8 874 eligible subjects, the response rate was of 80%, giving a final sample of 7 164 adults 60 years or older, who were included in this report. Personnel who had received standardized training did face-to-face interviews in the older adults’ homes. A proxy was used if the older person presented cognitive impairment.

### Measurements

The following conditions were considered in our research in order to test older adults’ health status: weight loss, disability, falls, depressive symptoms, and positive affectivity. Weight loss was considered present if the older adult responded affirmatively to the question, “In the last three months, have you lost weight?” Disability was considered present if the subject had difficulty or could not perform one or more activities of daily living, out of an eight-item list: walking in a room, bathing, getting in or out of bed, dressing, preparing a hot meal, shopping, taking medication, and managing money. Falls were considered present if the subject had fallen at least once in the preceding year. Depressive symptoms were assessed with a modified Spanish-language version of the Center for Epidemiologic Studies Depression Scale (CESD), with only six questions. A positive answer to four out of the six questions on depressive symptoms was considered as high depressive symptomatology, as already validated in Mexican older adults. A seventh question from this set was used to assess positive affectivity: “In the last week, I enjoyed life.” If the older adult answered negatively, it was considered as low positive affectivity. In order to assess socioeconomic status, a measurement referred to as the poverty composite index was used. It was defined as a high poverty composite index if the older adult lived in a community with fewer than 2 500 people and limited access to services.

Violence was assessed using the CIDAC index (*índice del Centro de Investigaci*ó*n para el Desarrollo, A.C.* [Research Center for Development]), a model that measures the impact that violence has on citizens. It includes eight different criminal offenses closely related to drug trafficking: kidnapping, homicide, stab wounds, extortion, robbery without violence, robbery with violence, carjacking without violence, and carjacking with violence. These specific offenses are important as far as the public perception of crime’s impact (24). Their occurrence is related to the belief of possibly becoming a victim of another crime in the future. The product of the multiplication of a given crime incidence by the estimated weight of that particular crime on the perception of insecurity was used to generate the final score. A score was given for each of the 32 states of Mexico, as reported elsewhere (24). A higher score was related to a lower perception of being a possible crime victim, with 100 being the highest possible score. In other words, the higher the score, the less people thought they would be victims of a crime. Lower scores were related to more violence, with zero being the lowest possible score. The outcome was a national ranking of crime perception categorized into four groups: moderate (> 87), medium (87–78), serious (77–68), and severe (< 68).

### Statistical analysis

Descriptive statistics were performed with the mean and standard deviations (SDs) for continuous variables with normal distribution. The median with interquartile range (IQR) was employed for abnormally distributed variables. For categorical variables stratified by sex, absolute and relative frequencies were used. Multiple logistic regressions with unadjusted and adjusted models (for age, sex, and poverty composite index) were performed. All analyses were run with Stata version 13.1 statistical software (StataCorp, College Station, Texas, United States of America).

### Ethical issues

The study was approved by the research and ethics committees of Mexico’s National Institute of Public Health, which is in charge of conducting and implementing the ENSANUT surveys and maintaining the public databases of the results. All the study subjects signed an informed consent form. We researchers did not perform any additional data collection, and our analysis was secondary. The National Institute of Public Health manages the data according to Mexico’s current federal law for protection of personal information. Under that law, protection of data in academic and research institutions is the responsibility of the main researcher, and he or she is the only one who has access to data to identity research subjects. Revealing this information is a felony, so data are saved in encrypted files that can be opened only under circumstances in which the benefit of revealing someone’s identity outweighs the concerns for anonymity.

## RESULTS

A total of 7 164 people were included in the analysis. The mean age was 70.6 (standard deviation (SD), 8.1), 70.8 in men (SD, 8.0) and 70.5 in women (SD, 8.1). There were 2 671 older adults living in highly marginal communities, 51.8% (*n =* 1 384) women and 48.2% (*n* = 1 287) men. The population studied fell into the following categories of the CIDAC violence index: 20.7% (*n* = 1 486) moderate; 26.1% (*n* = 1 870) medium; 26.6% (*n* = 1 904) serious; and 26.6% (*n* = 1 904) severe. Worse CIDAC violence categories were more frequent for women than for men (severe 56.6% for women versus 43.3% for men). Regarding health conditions, 35.0% (*n* = 2 511) of the sample had experienced a weight loss, especially women 56.4% (*n* = 1 416) versus men 43.6% (*n* = 1 095). The total percentage of participants with a disability was 16.9% (*n* = 1 212); 62.7% (*n* = 761) of these were women. Women reported a higher percentage of falls (60.5%, *n* = 1 545) compared to men (39.4%, *n* = 1 007). One-third of the sample had depression (*n* = 2 432); 64.1% (*n* = 1 559) of these were women. Low positive affectivity was reported in 31.1% of the total sample (*n* = 2 230), with a higher percentage found among women (59.7%, *n* = 1 333). A high percentage of participants (80.2%, *n* = 5 751) reported having at least one of the five health problems studied (weight loss, disability, falls, depression, or low positive affectivity). From these, 57.1% *(n* = 3 281) were women and 42.9% *(n* = 2 470) were men (Table 1).

**TABLE 1.** General characteristics of the population in the study of collective violence and the health of the elderly in Mexico in 2012, stratified by gender^a^, ^b^

Characteristic	Men (*n* = 3 241)	Women (*n* = 3 923)	Total (*N* = 7 164)
Age (mean and standard deviation)	70.8 (8.0)	70.5 (8.1)	70.6 (8.1)
High poverty composite index (*n* and %)	1 287 (48.2)	1 384 (51.8)	2 671 (37.2)
CIDAC violence level category (*n* and %)			
Moderate	903 (48.2)	967 (51.7)	1 486 (20.7)
Medium	671 (45.1)	815 (54.8)	1 870 (26.1)
Serious	842 (44.2)	1 062 (55.7)	1 904 (26.6)
Severe	825 (43.3)	1 079 (56.6)	1 904 (26.6)
Weight loss (*n* and %)	1 095 (43.6)	1 416 (56.4)	2 511 (35.0)
Disability (*n* and %)	451 (37.2)	761 (62.7)	1 212 (16.9)
Falls (*n* and %)	1 007 (39.4)	1 545 (60.5)	2 552 (35.6)
Depression (*n* and %)	873 (35.9)	1 559 (64.1)	2 432 (33.9)
Low positive affectivity (*n* and %)	897 (40.2)	1 333 (59.7)	2 230 (31.1)
Any problem (*n* and %)	2 470 (42.9)	3 281 (57.0)	5 751 (80.2)

**Source:** Produced by the authors from the study data.

a The results shown in the table are based on data from the ENSANUT 2012 (Encuesta Nacional de Salud y Nutrición [Mexican National Survey on Health and Nutrition]) and a violence index developed by the Centro de Investigación para el Desarrollo, A.C. (CIDAC) [Research Center for Development].

b The percentages shown for each gender correspond to the respective lines and thus are for informative purposes, not for comparative purposes.

As shown in Table 2, the multiple logistic regression of the CIDAC violence index categories (according to the state’s score, which was considered as an independent variable) and geriatric health issues showed that weight loss had an incremental association with the categories when compared with the reference category (moderate). When the model was adjusted for age, sex, and high poverty composite index, this association was unchanged for medium weight loss (odds ratio (OR), 1.17; 95.0% confidence interval (CI), 1.12–1.35; *P =* 0.027); for serious weight loss (OR, 1.20; 95.0% CI, 1.05–1.38; *P =* 0.007); and for severe weight loss (OR, 1.20; 95.0% CI, 1.05–1.40; *P =* 0.006). There was no association for disability and falls. Depressive symptoms were only associated when contrasting the severe category to the reference category (OR, 1.14; 95% CI, 1.01–1.30; *P =* 0.047) in the unadjusted model, and losing significance when the adjusted model was used. Low positive affectivity was found significantly associated in both the unadjusted model and the adjusted model, but only when contrasting the severe category with the reference category (OR, 1.21; 95% CI, 1.06–1.40; *P* = 0.005); the other categories lost significance in the adjusted model. Finally, using the adjusted model, having any geriatric health problem was associated when the reference was compared with the serious category (OR, 1.20; 95% CI, 1.02–1.45; *P =* 0.029) and the severe category (OR, 1.19; 95% CI, 1.01–1.43; *P =* 0.049).

**TABLE 2.** Multiple logistic regression with unadjusted and adjusted models for geriatric problems and CIDAC^a^ violence index groups in the study of collective violence and the health of the elderly in Mexico, 2012, with odds ratios (ORs) and 95% confidence intervals (CIs)

Geriatric problem and CIDAC violence index groups	Unadjusted OR (95% CI)	*P* value^b^	Adjusted^c^ OR (95% CI)	*P* value^b^
Weight loss			
Moderate	Reference		Reference	
Medium	1.17 (1.01–1.30)	.032	1.17 (1.02–1.35)	.027
Serious	1.19 (1.10–1.40)	.009	1.20 (1.05–1.38)	.007
Severe	1.21 (1.06–1.40)	.005	1.20 (1.05–1.40)	.006
Disability				
Moderate	Reference		Reference	
Medium	.92 (.80–1.07)	.315	.93 (.80–1.09)	.408
Serious	.94 (.82–1.08)	.413	.96 (.83–1.10)	.571
Severe	.89 (.78–1.0)	.118	.86 (.75–1.0)	.052
Falls				
Moderate	Reference		Reference	
Medium	.95 (.82–1.09)	.501	.94 (.82–1.09)	.463
Serious	.96 (.84–1.10)	.562	.95 (.83–1.09)	.503
Severe	1.05 (.92–1.20)	.444	1.03 (.90–1.17)	.647
Depression				
Moderate	Reference		Reference	
Medium	1.09 (.90–1.20)	.222	1.06 (.92–1.23)	.378
Serious	1.14 (.99–1.30)	.051	1.11 (.96–1.27)	.135
Severe	1.14 (1.01–1.30)	.047	1.11 (.97–1.28)	.110
Low positive affectivity				
Moderate	Reference		Reference	
Medium	1.15 (1–1.34)	.050	1.14 (.98–1.32)	.078
Serious	1.16 (1.01–1.3)	.034	1.14 (.99–1.31)	.063
Severe	1.23 (1.10–1.41)	.003	1.21 (1.06–1.4)	.005
Any problem				
Moderate	Reference		Reference	
Medium	1.07 (.89–1.3)	.414	1.1 (.91–1.32)	.310
Serious	1.18 (1.10–1.41)	.050	1.2 (1.02–1.45)	.029
Severe	1.22 (1.02–1.4)	.023	1.19 (1.01–1.43)	.049

a CIDAC = *Centro de Investigaci*ó*n para el Desarrollo, A.C.* [Research Center for Development].

b *P* values were obtained from chi-square tests for categorical variables and from t-tests for continuous variables.

c Adjusted for age, sex, and high poverty composite index.

## DISCUSSION

To our knowledge, this is the first report that links collective violence to the health status of older adults in Mexico, a country with high violence rates due to the war on drugs. The incremental association of weight loss with the CIDAC violence index categories may be explained, in part, by a shortage of resources, and reduced food security, which is common in the elderly Mexican population, as shown by a recent report (25). It is possible that a worse perception of violence is related to a person’s ability to leave the house to shop for food and to socialize. A direct association between nutritional risk and isolation due to negative social support and negative social capital (in this case, reflected by collective violence) has been shown to be stronger among minorities (26). In addition to weight loss, positive affectivity of older adults was also affected in the states with the highest violence indices, which could impact on the progression to high depressive symptomatology, as shown in a study conducted in Mexico on the trajectories of depressive symptoms in older adults (27). Moreover, the composite variable of any geriatric health problem showed a significant association with the higher categories of violence. This may mean that the health of older adults, when analyzed comprehensively, is affected by collective violence. In fact, this could be a reflection of the intricate interaction between social determinants and the inner complexity of an aging individual.

The CIDAC index gives an approximation of how the Mexican population perceives violence. However, the index does not take into account the level to which people have become accustomed to living in a violent setting. For example, it may not make allowance for how collective violence could also impact domestic violence (specifically violence against older adults), which in turn could worsen the overall health status of an older adult.

Social vulnerability is the degree to which a person’s overall social situation leaves that person susceptible to health problems (28). Lack of social support, which is common in violent settings, is among the social factors that influence health, as is socioeconomic status (29).

Although it is known that living in a violent environment affects individuals and families (30), literature on the impact of collective violence on health is mainly focused on children and teenagers. Reports on adults are centered mostly on mental health repercussions, showing posttraumatic stress disorder and major depression to be associated with violence (31). Therefore, having a wider view of the impact of collective violence on all the members of a given family should be a part of the assessment of older adults (or any other age group).

As noted previously, research on violence among the elderly is focused on domestic violence and abuse. Those two topics are usually explored as entities that revolve around families, as if families were isolated from the social environment. It has been shown that living in a violent household can be prejudicial to health, as is living in an environment that is extremely violent (as perceived by the inhabitants) (30).

Conflict is prevalent all around the world, and we are all exposed to violence to some degree. It is important that the detrimental effects that violence has on health be addressed as a global health issue. A first step is to measure the perception of violence as part of the assessment of older adults, in particular those living in violent settings (e.g., where there is organized crime, terrorism, and war) (31). This should be done routinely along with screening for domestic violence and abuse in a geriatric health care context. Examining the indirect effects of violence in this specific age group are relevant for social and health systems.

As is the case with many countries in Latin America, high levels of organized crime have existed in Mexico for several decades. However, it is undeniable that the level of violence has increased in recent years (5, 31). The effect of this situation on the health of the Mexican population is still not fully known. As already stated by Gamlin (5), we believe that violence should be studied as part of national health surveys and in clinical settings, so as to understand the real impact it is having on the health of adults over 60 years old living in places with ongoing collective violence. Knowledge of the magnitude of the problem will allow for strategies to be planned to decrease violence and its effects on health. The Government of Mexico must take this issue seriously and must be held accountable for the consequences of the war on drugs (and other serious consequences of organized crime).

Framing violence in an explanatory context points to the fact that no simple solutions are available, and that attention has been lacking on the issue of how collective violence and individual health are related (11). To provide a definite solution to this vicious circle, it will be necessary to both consider the direct consequences of collective violence and to shift the focus on how violence is thought to affect health indirectly.

The limitations of our study are mainly related to the integration of the state-level CIDAC scale of perceived violent crime versus the individual analysis of health variables as presented in ENSANUT. Defining violence only as collective violence in framing our study and using the CIDAC index leaves out other definitions and concepts that might be related. In addition, the CIDAC index only considers some criminal offenses, therefore limiting the data and the analysis. Another limitation of our study was that it was not possible to know whether weight loss was the consequence of a previous state of overweight or underweight.

The strengths of our study, as stated before, include our approach to two very important, challenging issues in public health—older adults and violence—and their relationship. The use of an index created outside the health field supports the multidisciplinary strategy necessary for this type of study.

## Conclusions and recommendations

Our results show that collective violence is associated with weight loss and other health problems. Collective violence could indirectly affect individuals’ health, especially among vulnerable groups, such as older persons.

Mexico is just one of many countries enduring violence as a way of life. We encourage other investigators to conduct research around older adults and collective violence. The results of that work could be translated into public health policies and even arguments to end collective violence. We also urge public health policymakers to turn some of their efforts towards older adults, who make up one of the most vulnerable populations. Finally, in settings where collective violence is commonplace, assessing the impact of such violence on older adults’ health should become a routine activity for the health care system.

### Disclaimer.

Authors hold sole responsibility for the views expressed in the manuscript, which may not necessarily reflect the opinion or policy of the *RPSP/PAJPH* or PAHO.
